# Supplementary material for: Daytime SHP2 inhibitor dosing, when immune cell numbers are elevated, shrinks neurofibromas
Source: Life Sci Alliance. 2025 Sep 24;8(12):e202503359. doi: 10.26508/lsa.202503359 (PMC12461152; doi:10.26508/lsa.202503359)
Supplement: Supplementary file 1 [file LSA-2025-03359_SdataF1.pptx]

## Slide 1
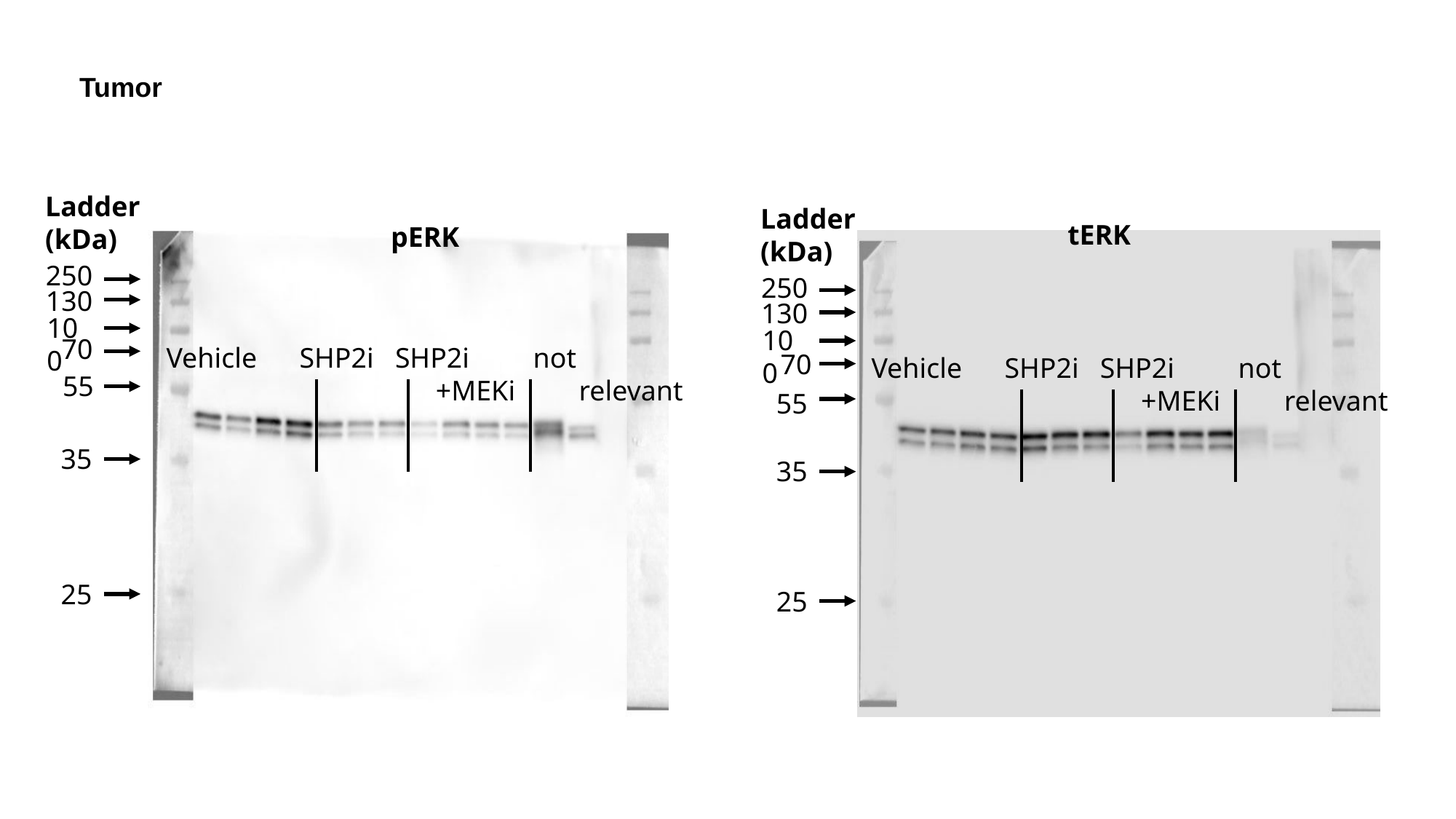

Tumor
Ladder
(kDa)
250
130
100
70
55
35
25
Ladder
(kDa)
250
130
100
70
55
35
25
tERK
pERK
Vehicle SHP2i SHP2i not
 +MEKi relevant
Vehicle SHP2i SHP2i not
 +MEKi relevant

## Slide 2
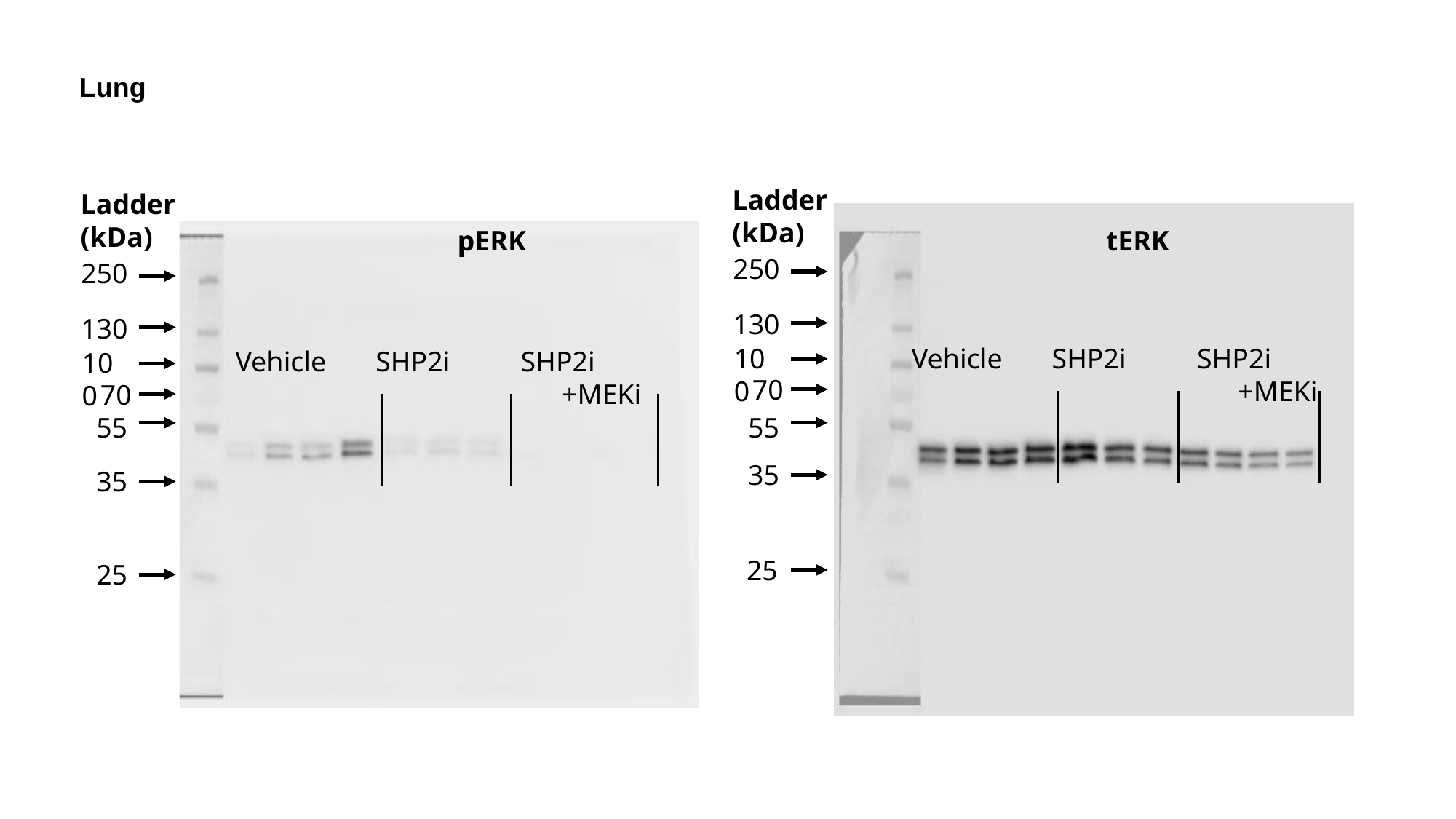

Lung
Ladder
(kDa)
250
130
100
70
55
35
25
Ladder
(kDa)
250
130
100
70
55
35
25
pERK
tERK
Vehicle SHP2i SHP2i
 +MEKi
Vehicle SHP2i SHP2i
 +MEKi
